# Catastrophic Case of West Nile Virus Rhombencephalitis in AIDS

**DOI:** 10.1177/23247096241267132

**Published:** 2024-07-30

**Authors:** Rupam Sharma, Elika Salimi, Carlos D’Assumpcao, Michael Valdez, Akriti Chaudhry, Arash Heidari, Rasha Kuran, Janpreet Bhandohal

**Affiliations:** 1 Kern Medical, Bakersfield, CA, USA; 2 David Geffen School of Medicine at UCLA, Los Angeles, CA, USA; 3 Western University of Health Sciences, Pomona, CA, USA

**Keywords:** West Nile virus rhombencephalitis, West Nile virus in an AIDS patient, neurosyphilis, and West Nile virus

## Abstract

West Nile Virus (WNV) belongs to the Flaviviridae family of viruses. It was first isolated and identified in 1937. Patients typically present with flu-like symptoms or are asymptomatic; however, neuroinvasive West Nile can lead to significant neurological impairment. Herein presented is a catastrophic case of WNV rhombencephalitis in a male patient newly diagnosed with AIDS. This report sheds light on the potential for severe neurological complications in co-infected patients and emphasizes the importance of early recognition.

## Introduction

West Nile Virus (WNV) is a single-stranded RNA virus member of the *Flavivirus* genus that is primarily transmitted by the *Culex* mosquito.^1^ Human infections range from asymptomatic to neurological devastation. At present, there is no established treatment available. The Center for Disease Control and Prevention (CDC) provides case reports of steroids, intravenous immunoglobulins (IVIGs), and interferon alpha 2b for treatment.^2^ We present a debilitating case of WNV rhombencephalitis in a person newly diagnosed with AIDS. Diagnostic and treatment challenges are discussed.

## Methods

This study was approved by the Institutional Review Board of Kern Medical. A retrospective review of both the patient’s records was performed. Literature search was conducted on PubMed and Google Scholar. The following search terms were applied: WNV rhombencephalitis, WNV in an AIDS patient, neurosyphilis, and WNV.

## Case Presentation

A 26-year-old homeless male, with no known medical history, presented to our institution with right arm weakness that started the morning of the presentation. This was associated with fever, night sweats, and unintentional weight loss. Two weeks prior to the presentation, the patient endorsed intermittent headaches which were relieved by ibuprofen. One week prior to the presentation, the patient stated his right arm started to feel weak. One day prior, the patient stated his weakness worsened and has issues walking. Per his mother, he had been displaying mild personality changes (more aggressive and moodier).

**Image 1.**
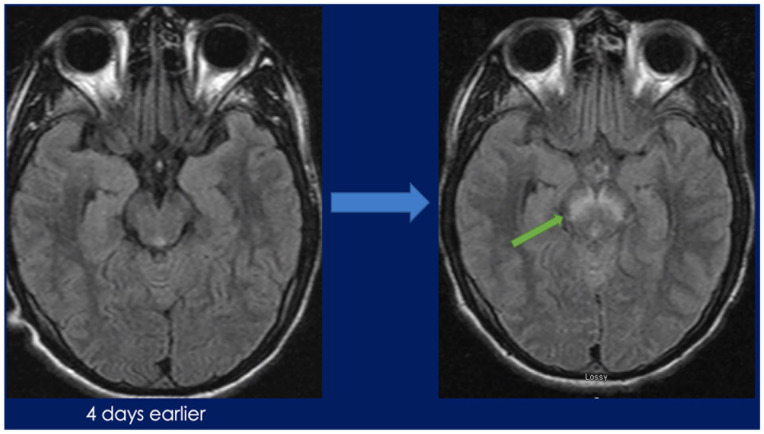
MRI brain at admission (left) and 4 days after admission (right).

**Image 2.**
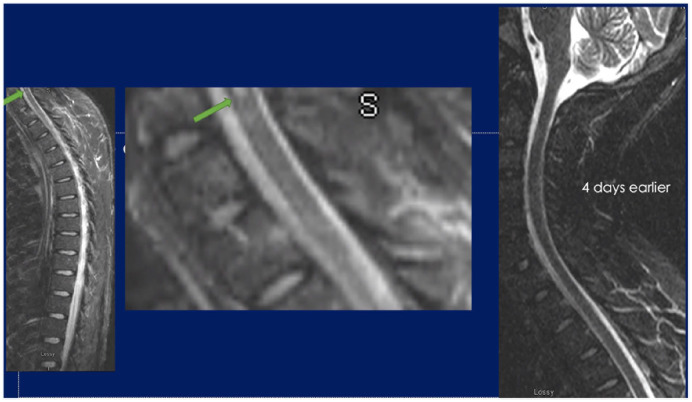
MRI revealing rhombencephalitis with displayed increased FLAIR and T2 signals involving the lower brainstem at the level of cerebellar peduncles and upper cerebellum in symmetric fashion compared to initial MRI at admission (right).

He developed waxing and waning mentation during his stay in the emergency department. Initial vitals were notable for a fever of 39.4°C and tachycardia with heart rate of 120 beats per minute. On physical examination, he was found to have flattening of the right nasolabial fold, stiff neck, and rotatory nystagmus when looking to the right. He could not lift his right arm and was found to have 1 out of 5 strength in his right deltoid and 4 out of 5 strength in his right biceps. A lumbar puncture was completed due to high-grade fever and revealed an opening pressure of 15 cm of water, with a white count of 320/mcL (red blood cell [RBC] 3, 10% neutrophils, 85% lymphocytes), glucose 51 mg/dL, and syphilis antibody qualitative returned positive. Empirical intravenous penicillin for the treatment of presumptive neurosyphilis was started. Shortly after his fluorescent treponemal antibody absorption (FTA-ABS) resulted positive, with rapid plasma regain (RPR) titer of 1:16, and venereal disease research laboratory (VDRL) titer of 1:1 in his cerebrospinal fluid (CSF). The following day, he was found to have continued right arm weakness and clonus bilaterally in the ankles with 3+ beats. On hospital day 3, the patient developed worsening mentation, hyperreflexia, status epilepticus, absent corneal and gag reflex, right rotatory nystagmus with respiratory failure, and was subsequently intubated. A repeat magnetic resonance imaging (MRI) brain on day 4 revealed rhombencephalitis with displayed increased FLAIR and T2 signals involving the lower brainstem at the level of cerebellar peduncles and upper cerebellum in symmetric fashion (Images 1 and 2). In addition, HIV antigen/antibody and quantitative polymerase chain reaction (PCR) resulted positive with viral load of 19 000/mL and CD4 count of 44 mcL. Empirical ampicillin for listeria and ganciclovir for cytomegalovirus (CMV) and herpes simplex virus (HSV) was started until CSF multiplex PCR was negative. Rifampin, isoniazid, pyrazinamide, and ethambutol were added empirically as well. After consideration of risk of immune reconstitution syndrome and benefit of antiretroviral therapy, he was initiated on tenofovir disoproxil fumarate, emtricitabine, and dolutegravir crushed into an orogastric tube. Furthermore, into his hospitalization, he developed intractable seizures requiring combinations of 5 antiepileptic medications. Cerebrospinal fluid and serum WNV IgM and IgG returned negative. At this point, antituberculosis medications were stopped. He completed 7 days of dexamethasone, 5 doses of IVIGs, and a 14-day intravenous (IV) Penicillin G treatment without meaningful improvement. Overall, patient was treated for HIV, syphilis, and WNV. A tracheostomy and percutaneous gastrostomy tube were placed on hospital day 18. Cerebrospinal fluid and serum WNV qualitative PCR returned positive on hospital day 20. Seizures abated on medications. After hospital day 30, the patient remained in a coma with minimal brain stem reflexes, and the patient was discharged to a long-term acute care facility. He subsequently passed away a few months later.

## Discussion

West Nile virus was first isolated and identified in 1937 in the West Nile district of Uganda from the blood of a febrile woman.^3^ The virus belongs to the Flaviviridae family, which also includes viruses like dengue, yellow fever, and Zika. Mosquitoes of the *Culex* genus, particularly *Culex pipiens* and *Culex quinquefasciatus*, were identified as the primary vectors responsible for transmitting the virus to humans and animals. Mosquitoes played a crucial role in the virus’s transmission as they fed on infected birds and then transmitted the virus to other birds, animals, and humans through subsequent bites.^3,4^ For many years, WNV was primarily found in Africa, the Middle East, and parts of Europe. In 1999, an outbreak of severe neurological illness occurred in New York City. Birds, particularly crows, were observed to be dying in large numbers, and it was soon discovered that the cause was WNV. This marked the first recorded outbreak of WNV in North America.^1,4^ The virus subsequently spread across the United States and into Canada, causing numerous human cases and deaths.^5^ Since then, WNV has become endemic in many parts of North America, causing seasonal outbreaks during the warmer months when mosquito activity is high. It has also been reported in various other countries around the world.^3,5^

Uncomplicated West Nile fever typically presents with flu-like symptoms or asymptomatic; however, neuroinvasive West Nile can lead to significant neurological impairment. Neuroinvasive West Nile disease occurs in <1% of the cases. It can manifest as encephalitis, meningitis, or poliomyelitis-like acute flaccid paralysis.^6^ It has been found that 50% to 71% of neuroinvasive WNV patients develop encephalitis which usually involves muscular weakness and malfunction of the peripheral nervous system.^7^ Moreover, 10% to 30% of these patients lead to their demise.^6^ West Nile rhombencephalitis is a rare but potentially serious manifestation of WNV infection. Rhombencephalitis refers to inflammation and involvement of the hindbrain, which includes the brainstem and the cerebellum. Symptoms of West Nile rhombencephalitis can include severe headache, high fever, neck stiffness, disorientation, tremors, muscle weakness, difficulty swallowing, and problems with balance and coordination.

The diagnostic criterion per the 2003 American Medical Association for WNV encephalitis includes encephalopathy (depressed or altered level of consciousness, lethargy, or personality change) for over 24 hours and having 2 or more of the following: fever (≥38°C) or hypothermia (≤35°C), CSF pleocytosis of >5 leukocytes/mm^3^, peripheral leukocyte count of >10 000/mm^3^, neuroimaging findings consistent with acute inflammation or acute demyelination, presence of focal neurological deficit, menigismus, electroencephalography (EEG) findings consistent with encephalitis, and seizures (either new or exacerbation of existing controlled seizure).^3,8^ In our case, the patient displayed all the following criteria except for the peripheral leukocyte count of >10 000/mm^3^ which was due to his HIV status. MRI suggesting rhombencephalitis displayed increased FLAIR and T2 signals involving the lower brainstem at the level of cerebellar peduncles and upper cerebellum in symmetric fashion, and EEG findings showing focal left frontotemporal status epilepticus (patient did not have a history of seizures).

West Nile Virus studies have shown that neuronal damage occurs from virus infection of neurons ranging from the chemoreceptor in the ventral medulla to the phrenic in the cervical cord.^9^ Our patient lost spontaneous movement of his limbs by the end of his hospital course and did not withdraw to pain. Damage of the cholinergic motor neurons in the lumbosacral spinal cord likely also impairs electrophysiological function of the neurons themselves, reducing the number of the functional motor units of hind limbs, leading to eventual paralysis.^10^

West Nile virusr hombencephalitis has a poor prognosis. The presence of this disease and delay in diagnosis was largely due to the presence of HIV. HIV mainly reduces CD4+ T-cells, and these cells are crucial in providing protection by maintaining antibody production and fostering WNV-specific CD8+ T-cell responses.^11^ CD8+ T-cells aid in clearing infection and preventing viral persistence in tissues, in this case, the central nervous system (CNS).^12^ This patient’s deterioration with WNV seemed to be related to his HIV T-cell deficiency. West Nile Virus encephalitis and B-cell deficiencies have been reported in the past such as a case of a patient after a lung transplant requiring rituximab, a CD20(+) monoclonal antibody (B-cell marker), who developed rapid, fulminant WNV meningoencephalitis within 6 months.^13^ However, no cases of WNV encephalitis due to T-cell deficiency have been reported to date.

While people of all ages are at risk of infection with WNV, the risk of advancement to neurological symptoms increases with age (over 60 years old) as well as with co-morbid diseases. This case, a patient at 26 years of age, was complicated by the presence of HIV, allowing WNV to thrive as well as delaying our diagnosis. Our patient with a CD4+ count of 44 cells/mcL, AIDS stage 3, had an immensely impaired immune system, unable to keep up with the rapid spread of WNV, and causing a seronegative result, specifically a negative CSF and serum WNV IgM and IgG. West Nile Virus serum IgM test has a sensitivity of 95% within 7 days (not diagnostic in CSF as it does not cross the blood-brain barrier). However, PCR testing with a sensitivity of 57% in CSF and 14% in serum, may have better sensitivity in immunodeficient patients due to the lack of mounting an adequate antibody response, prolonged viremia, and B-cell depletion.^3^

Although our patient did meet diagnostic criteria for West Nile rhombencephalitis, the patient’s initial serology and diagnostic testing including MRI resulted negative. It was not until hospital day 4 that a repeat MRI showed signs of rhombencephalitis. Once the new MRI findings came to light, a repeat lumbar puncture was done, and this time, all differential diagnosis for causes of acute rhombencephalitis needed to be accounted for. Therefore, *Listeria*, *Enterovirus*, and HSV 1 and 2 as well as CMV, and human herpesvirus 6 (HHV6) CSF panels were ordered. All came back negative on hospital day 14. Ultimately West Nile CSF and serum RNA reverse transcription (RT)-PCR came back positive, but this was not until hospital day 20, after multiple trials of treatment with other explanations of meningitis/encephalitis as mentioned above and tuberculosis, as well as co-treatment with penicillin-G were attempted. With WNV rhombencephalitis having a poor prognosis, the patient continued to deteriorate.

*Treponema pallidum*, a spirochete responsible for syphilis, also known as the “great imitator,” can damage the nervous system, cardiovascular, and skeletal system. It is known that HIV and syphilis increase the risk of being infected with the other and therefore anyone diagnosed with one, should be screened for the other.^14^ In fact, it has been reported that HIV/AIDS patients are 77 times more likely to contract syphilis than HIV-negative patients.^15^ In turn, this co-infection can increase the likelihood of developing neurosyphilis which our patient could have ended up with; however, neurosyphilis did not explain all our symptoms together.^16^ The patient also did not show signs of improvement with a completed 14-day course of IV penicillin G, and in fact, there have been no reported cases of rhombencephalitis from neurosyphilis. Meanwhile, cases of rhombencephalitis from WNV have been reported, and our patient did ultimately test positive for this, making WNV the most likely explanation.

In cases of severe WNV infection, with significant neurological involvement or inflammation, dexamethasone may be used to help reduce brain inflammation and alleviate symptoms. At the same time, IVIG has been used for neurological involvement of WNV as well. Intravenous immunoglobulin is a blood product containing pooled antibodies from multiple donors. The rationale for the treatment of WNV encephalitis is to provide passive immunity and modulate the immune response against the virus. However, the use of dexamethasone and IVIG in the treatment of WNV infection is still an area of ongoing research. Currently, research indicates that IVIG demonstrates the most favorable outcomes in the treatment of WNV encephalitis.^17,18^ Today, many vaccines against WNV in both humans and animals are in different developmental stages.^19^ However, none have been approved yet.

West Nile virus rhombencephalitis has a poor prognosis with fatalities up to 30% in combination with delayed diagnosis due to HIV-mediated seronegative results. To date, there are no licensed antivirals or treatment for WNV. If a patient meets criteria for CNS WNV, immunosuppression, and reliability of serology testing should be considered while pursuing PCR testing of serum and CSF. Given the ongoing trends of globalization and climate change, *Flavivirus* infections have escalated from being solely a matter of public health concern to a significant global health issue. A larger emphasis on the OneHealth Initiative uniting human and veterinary medicine on this matter is crucial.
